# Pythoscape: a framework for generation of large protein similarity networks

**DOI:** 10.1093/bioinformatics/bts532

**Published:** 2012-09-08

**Authors:** Alan E. Barber, Patricia C. Babbitt

**Affiliations:** Department of Bioengineering and Therapeutic Sciences, University of California, San Francisco, CA 94158, USA

## Abstract

Pythoscape is a framework implemented in Python for processing large protein similarity networks for visualization in other software packages. Protein similarity networks are graphical representations of sequence, structural and other similarities among proteins for which pairwise all-by-all similarity connections have been calculated. Mapping of biological and other information to network nodes or edges enables hypothesis creation about sequence–structure–function relationships across sets of related proteins. Pythoscape provides several options to calculate pairwise similarities for input sequences or structures, applies filters to network edges and defines sets of similar nodes and their associated data as single nodes (termed representative nodes) for compression of network information and output data or formatted files for visualization.

**Contact:** babbitt@cgl.ucsf.edu

**Supplementary information:** Supplementary data are available at *Bioinformatics* online.

## 1 INTRODUCTION

The rapid growth of databases of protein information (e.g. sequences and structures) provides both new opportunities and challenges for analysis and clustering by similarity. For example, global analysis of entire superfamilies and association of their members with biological information and other types of metadata has become a useful tool for functional annotation and discovery (Brown and Babbitt, 2012). As these sets become larger (sometimes many thousands of sequences) and their members more divergent, their fast exploration on a large-scale becomes less feasible using traditional approaches such as alignments and trees.

Protein similarity networks (PSNs) enable analysis and visualization of structure–function relationships in large protein data sets by clustering of individual protein sets for more complex analysis while summarizing ‘connectivity’ relationships among the clusters. Mapping orthogonal sources of biological information onto PSNs then provides a powerful way to view functional trends across the set that can be interpreted in the context of their similarities. (See Atkinson *et al.*, 2009 for an initial analysis of some uses and statistical validation of PSNs.)

While databases like Similarity Matrix of Proteins (SIMAP) (Rattie *et al.*, 2010) store pairwise similarities, and plug-ins available with software such as Cytoscape (Smoot *et al.*, 2011) allow creation of small PSNs (Wittkop *et al.*, 2010), no software solution exists to create and manage large PSNs. And while PSNs are inherently amenable to association with orthogonal information sources, the many information types available complicate development of a single software solution for managing such diverse features. Pythoscape addresses these issues and provides a software framework to create PSNs and develop new analyses for inference of functional properties in proteins.

## 2 DESCRIPTION AND SIGNIFICANCE

Pythoscape is an extensible computational framework implemented in Python to generate and analyze PSNs. For the user interested in generating large networks, the Pythoscape package has a core set of plug-ins (Supplementary Table S1) and tutorials, so that no development is needed to create simple networks painted with useful metadata. For software developers, Pythoscape provides a framework for rapid modification along with well-documented application programming interfaces for development of additional plug-ins using new sources of metadata.

**Fig. 1.**
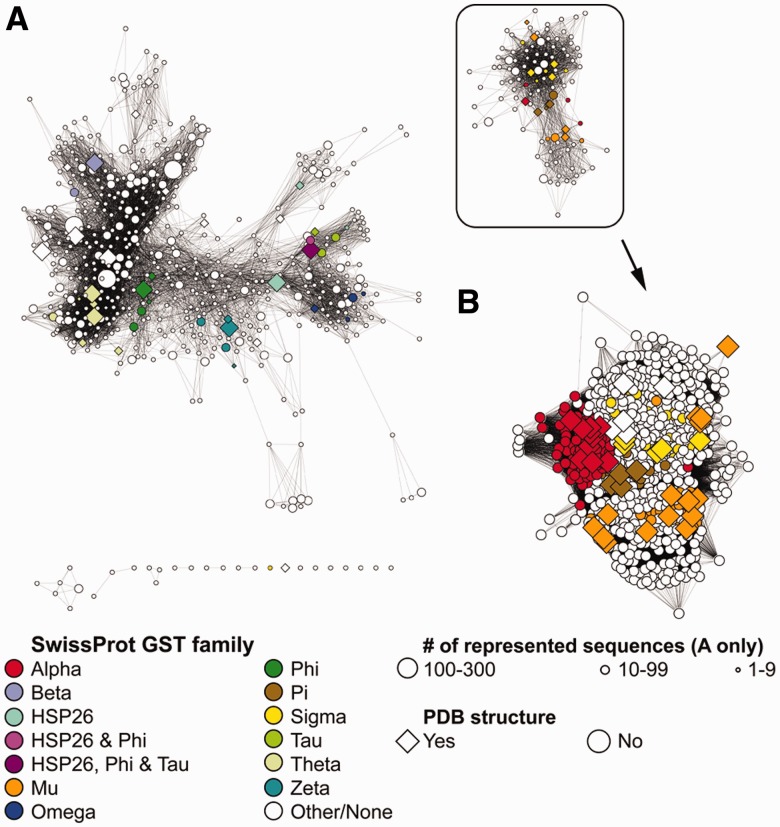
Sequence similarity network of the GST superfamily generated by Pythoscape and visualized in Cytoscape. To compact the view for this figure, networks were layed out using the organic layout in Cytoscape rather than the distances computed from a similarity metric. In all, 664 representative nodes are used to describe pairwise relationships among 7447 sequences. (**A**) Representative network with functional classes colored, if annotated by SwissProt in a family (The UniProt Consortium, 2011). Family membership is indicated if one or more sequences in the abstracted node are associated with that family. (**B**) Full non-abstracted network for the group of GSTs found mostly in eukaryotes (boxed in A)

Unlike sparser networks such as interaction networks, PSNs are frequently close to complete, often requiring storage and management of large quantities of data, and fast calculation (Supplementary Table S2). Pythoscape allows for flexible storage of data through the use of storage interfaces. Appropriate storage solutions can be chosen based on network size or developed as needed allowing for easy updating for faster and more reliable database software solutions. Pythoscape can create, store and manage large networks, then, using representative nodes and edges to compress the information, output smaller summary networks for visualization (Fig. 1A and B). Users can choose how distances between representative nodes are calculated and, importantly, the full set of sequences in each node is retained for later use.

Additionally, Pythoscape has plug-ins for creating structure similarity networks and for generating correlations for edge distances between networks generated from a set of sequences and a corresponding set of available structures (Supplementary Table S1 and Supplementary Figs. S2 and S3).

## 3 EXAMPLE USAGE

Glutathione transferases (GSTs) are enzymes that typically catalyze the addition of glutathione to substrate compounds. They play roles in many biological processes, including metabolism of endogenous compounds and xenobiotics such as drugs. Of the thousands of GSTs that have been identified, the physiological substrates of only a small proportion are known; thus, they are principally classified into putative functional classes according to enzymatic, structural, and other features (Mannervik and Danielson, 1988). Recently, PSNs have been used to summarize and guide a global interpretation of GST sequence and structure relationships (Atkinson and Babbitt, 2009).

A PSN of GST sequences is shown in Figure 1A (see supplementary information for network creation and graph statistics). It illustrates how representative nodes computed by Pythoscape enable analysis of PSNs too large to be visualized in total while retaining their value for developing hypotheses from sequence similarities across the whole set. For comparison, individual clusters of interest can be outputted with all nodes present (Fig. 1B). This full non-abstracted network (representing a node for each sequence) shows a similar pattern of relationships to those shown in the corresponding representative node network (boxed in Fig. 1A). The correlation between the ideal representative node mean distances calculated in Pythoscape and the corresponding full network ideal distance for Fig. 1A is provided in Supplementary Figure S1. A quantitative description of the relationships between filtered networks and full networks has also recently been described elsewhere for some example systems (Atkinson *et al.*, 2009), but these differences appear also to depend on the specific system analyzed. While ‘missing data’ is an inherent feature of representative nodes, the trade-off is in visualizing similarity relationships across large datasets that would not be practically achievable because of memory and speed limitations in their calculation.

The network shown in Figure 1A demonstrates another issue in the use of representative nodes that could complicate interpreting relationships between functional features and sequence similarity. In the example given here, some GST families are represented by multiple representative nodes, whereas other representative nodes contain multiple SwissProt families (HSP26, Phi and Tau), obscuring how sequence similarity tracks with annotation. Thus, we recommend that analysis using representative networks be accompanied by examination of the relevant parts of the corresponding full networks.

## 4 CONCLUSION

Pythoscape is a software framework to efficiently create and manage protein similarity networks. Tutorials, Pythoscape documentation, source code and future development plans are available at http://www.rbvi.ucsf.edu/trac/Pythoscape.

## Supplementary Material

Supplementary Data
