# Supplementary material for: Pythoscape: a framework for generation of large protein similarity networks
Source: Bioinformatics. 2012 Sep 8;28(21):2845–6. doi: 10.1093/bioinformatics/bts532 (PMC3476340; doi:10.1093/bioinformatics/bts532)
Supplement: Supplementary Data [file supp_28_21_2845__index.html]

Pythoscape: A framework for generation of large protein similarity networks — Pythoscape: a framework for generation of large protein similarity networks — Supplementary Data 

# Pythoscape: a framework for generation of large protein similarity networks

## Supplementary Data

files

**Files in this Data Supplement:**

- Supplementary Data - pdf file
