# Supplementary material for: Pythoscape: a framework for generation of large protein similarity networks
Source: Bioinformatics. 2012 Sep 8;28(21):2845–6. doi: 10.1093/bioinformatics/bts532 (PMC3476340; doi:10.1093/bioinformatics/bts532)
Supplement: Supplementary Data [file supp_bts532_SupplementaryInformation-Revised.pdf]

Supplementary Information

Network Creation and Graph Statistics

Pythoscape v1.01b was used to create and annotate the networks. The network depicted in figure 1A was generated using all full-length protein sequences from Pfam 24.0 GST, GST_N (PF02798) (Punta *et al.*, 2012) and contains 664 representative nodes for 7,447 sequences from the Pfam GST family and10,881 representative edges representing 2,125,783 edges. One representative node is depicted for each cluster of sequences defined by CD-HIT v4.5.6 (Li *et al.*, 2006) using a 40% sequence identity threshold. An indication of the number of sequences represented in each representative node is given in the legend. Similarity between representative nodes in figure 1 is calculated as the mean -log10(E-value) of all pairwise BLAST+ (Camacho *et al.*, 2009) alignments < 1 between all sequences abstracted by each representative node. Edges are shown if the mean -log10(E-value) for pairwise comparisons of the sequences within a representative node is greater than a threshold of 13; edges at this threshold represent alignments with a median 31% identity over 205 residues. Pythoscape allows the user to choose the mean, max or minimum edge as the representative edge score between two groups of represented nodes. All represented information is stored in the database so that the user can output selected sub-networks using the entire set of sequences in each.

The network depicted in figure 1B contains the full network from the boxed cluster in figure 1A. It contains 702 sequences and only edges with pairwise BLAST -log10(E-value) alignment score of greater than 13.

Both networks are visualized in Cytoscape v2.8.3 using the organic layout. This layout solely uses node connectivity to calculate layout position and not ideal distance. This results in a more compact network for viewing; as reported elsewhere, layout positions for PSNs using the organic layout are similar to those calculated using a force directed layout computed from ideal distances (Atkinson, H.J., J.H. Morris, *et al.*, 2009, supplementary information table 1 with Pearson's correlation (R) ranging from 0.838 to 0.924 for eight different systems). Nodes are colored according to SwissProt GST family classifications. Annotations for SwissProt GST family and PDB structures were taken from UniProt release 2012_07 (The UniProt Consortium, 2011). White nodes are those from the Pfam set that are not annotated in the SwissProt database, nor are these annotated with any of the main classes shown in the figure legend.

Supplementary Table 1. Pythoscape plug-ins currently provided.

Plug-in name	Type	Description
AddAttributesByGreatestSum	Input	Create representative node attributes by calculating the node attribute with the greatest count
AddAttributesByIfAny	Input	Create representative node attributes by concatenating all attributes in nodes represented
AddBLASTEdgesFromLocalBLAST	Input	Calculate BLAST edges
AddBLASTEdgesFromTableRun	Input	Add BLAST edges from a table file
AddEdgesToRepnodeNetwork	Input	Calculate representative edges
CalcNodeSize	Input	Calculate how many nodes are in a representative node
CalculateTMAlignScores	Input	Calculate structure similarity using TM-align
CreateCDHITRepnodes	Input	Use CD-HIT to cluster nodes for representative networks
DeleteEdgesFromFile	Delete	Delete edges as specified by a file
ImportAttributeTable	Input	Input attributes from a file
ImportFromFastaFile	Input	Input sequences from fasta file
ImportFromUniProt	Input	Import node attributes from UniProt
ImportIdentifierTable	Input	Input node identifiers from table file

OutputAttributeClusters	Output	Output attributes from representative nodes
OutputAttributeTable	Output	Output table file of node attributes
OutputIdentifierTable	Output	Output table of node identifiers
OutputCorrelation	Output	Output correlation plot between two edge attributes
OutputXGMML	Output	Output XGMML file for visualization
PDBImportFromCSVFile	Input	Import structures from PDB database into Pythoscape
SequenceTableRun	Output	Output set-up files for parallel sequence edge run
StructureTableRun	Output	Output set-up files for parallel structure edge run

Supplementary Table 2. Benchmarks for edge calculations using Bl2Seq from BLAST+ v2.2.25 and parallel_blast2seq.py from the Pythoscape package on a system with 8 Intel(R) Xeon(R) X7560s at 2.27GHz (64 cores available) and 129,004 MB RAM. Each run as performed using a single edge input file for the purposes of the benchmark. Further speedup is possible with larger edge runs by splitting the run into several input files. In practice, we have routinely used Pythoscape to create networks of up to 50,000 nodes with roughly a billion edges using a large, shared cluster.

# nodes (protein sequences)	# edges (Bl2Seq runs)	# threads used	time (seconds)	# edges/thread/second
10	45	2	4	5.625
10	45	4	2	5.625
10	45	8	1	5.625
100	4,950	2	323	5.625
100	4,950	4	153	7.662
100	4,950	8	77	8.088
1000	499,500	2	67072	3.724
1000	499,500	4	33525	3.725
1000	499,500	8	22184	2.815

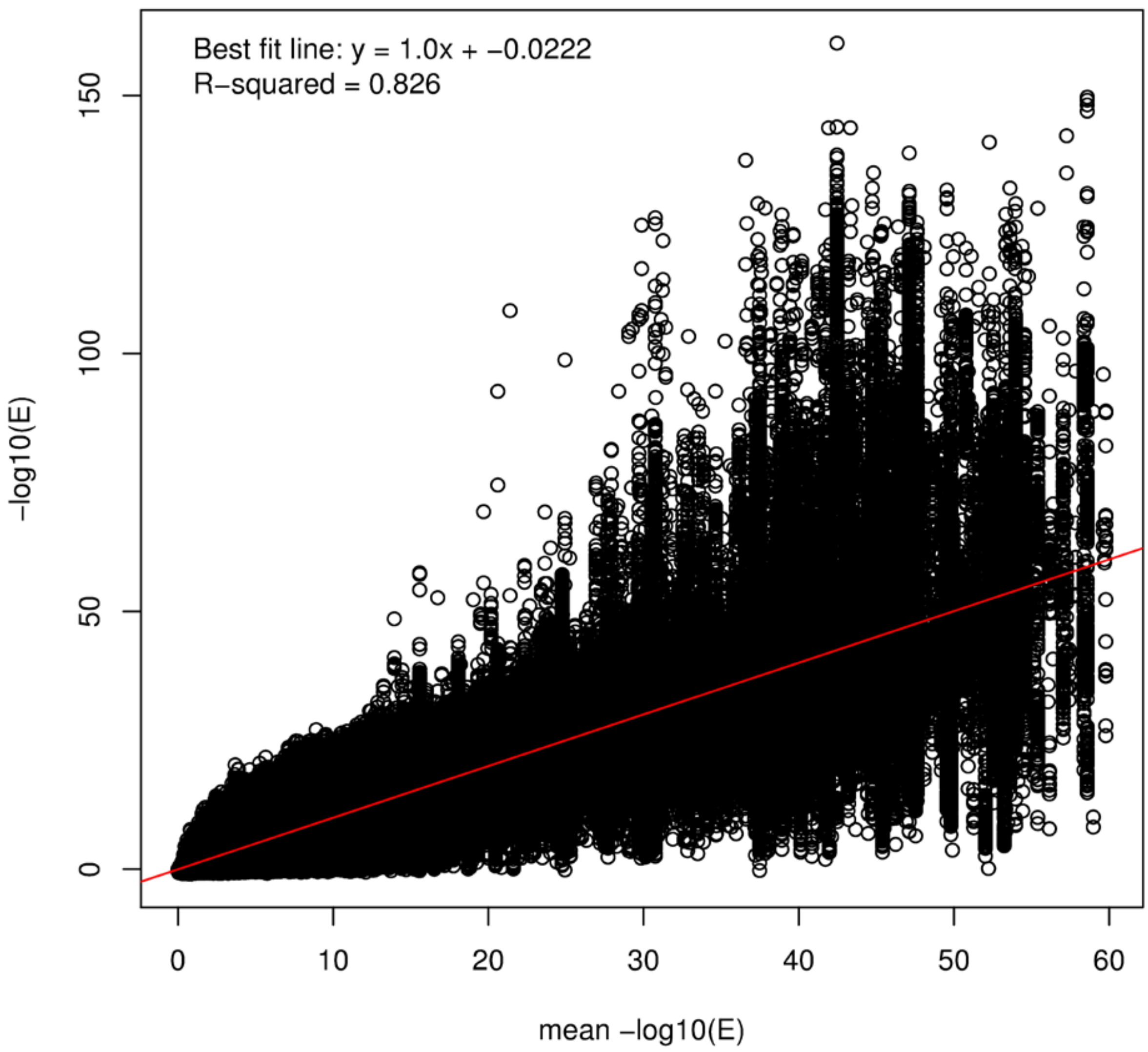

Supplementary Figure 1. Correlation between ideal representative –log10(Evalue) node distances and ideal full network –log10(Evalue) node distances for GST sequences. The plot of mean edge scores between representative nodes and their associated represented edge scores that both have an E-value < 1 and a mean –log10(E) < 60 shows a linear correlation. Outlier edges scores with mean –log10(E) > 60 were not shown as the scores are highly influenced by sequence length. Of 13,083,599 edge scores represented by the representative network used to create figure 1, 13,080,754 are plotted here. This figure was generated by the output_correlation.py Pythoscape plug-in.

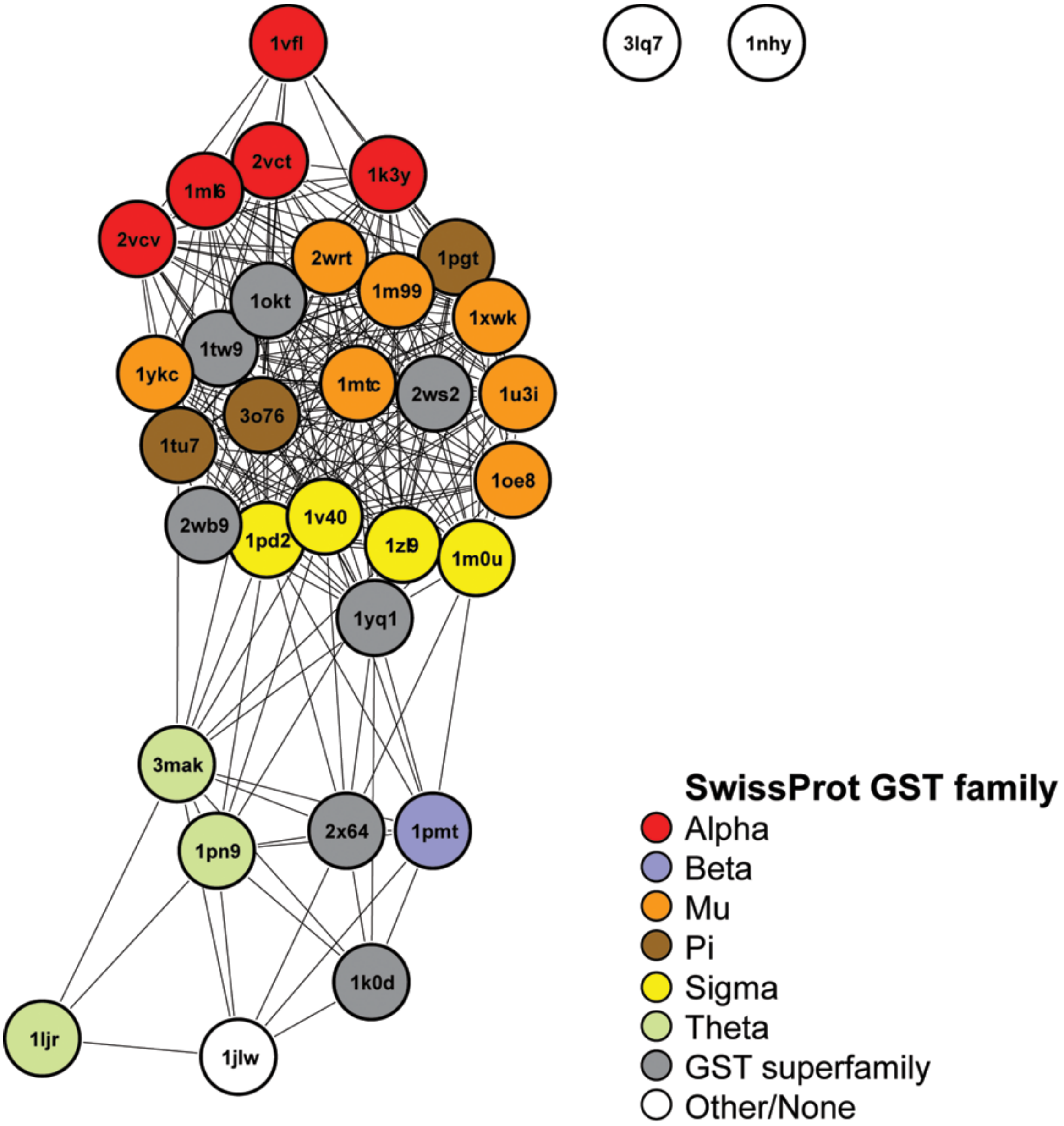

Supplementary Figure 2. Structure similarity network of GST structures colored by SwissProt GST family. Pairwise similarities for a non-redundant set of 33 structures from the Pfam 26.0 GST_N (PF02798) family were calculated using TM-align (Zhang *et al.*, 2005) Edges between nodes are drawn only if the TM-score > 0.80 (median number of aligned residues = 187; median RMSD = 2.32Å) for that edge. Structures are classified to SwissProt families as in figure 1. Each node is labeled by the PDB structure it represents. The network is visualized using the organic layout in Cytoscape. The correlation between edge distance for this structure similarity network and the corresponding –log10(E-value) edge in the sequence similarity network in figure 1A is plotted in supplemental figure 3. While the distances are similar, there is much less coverage in the structure network.

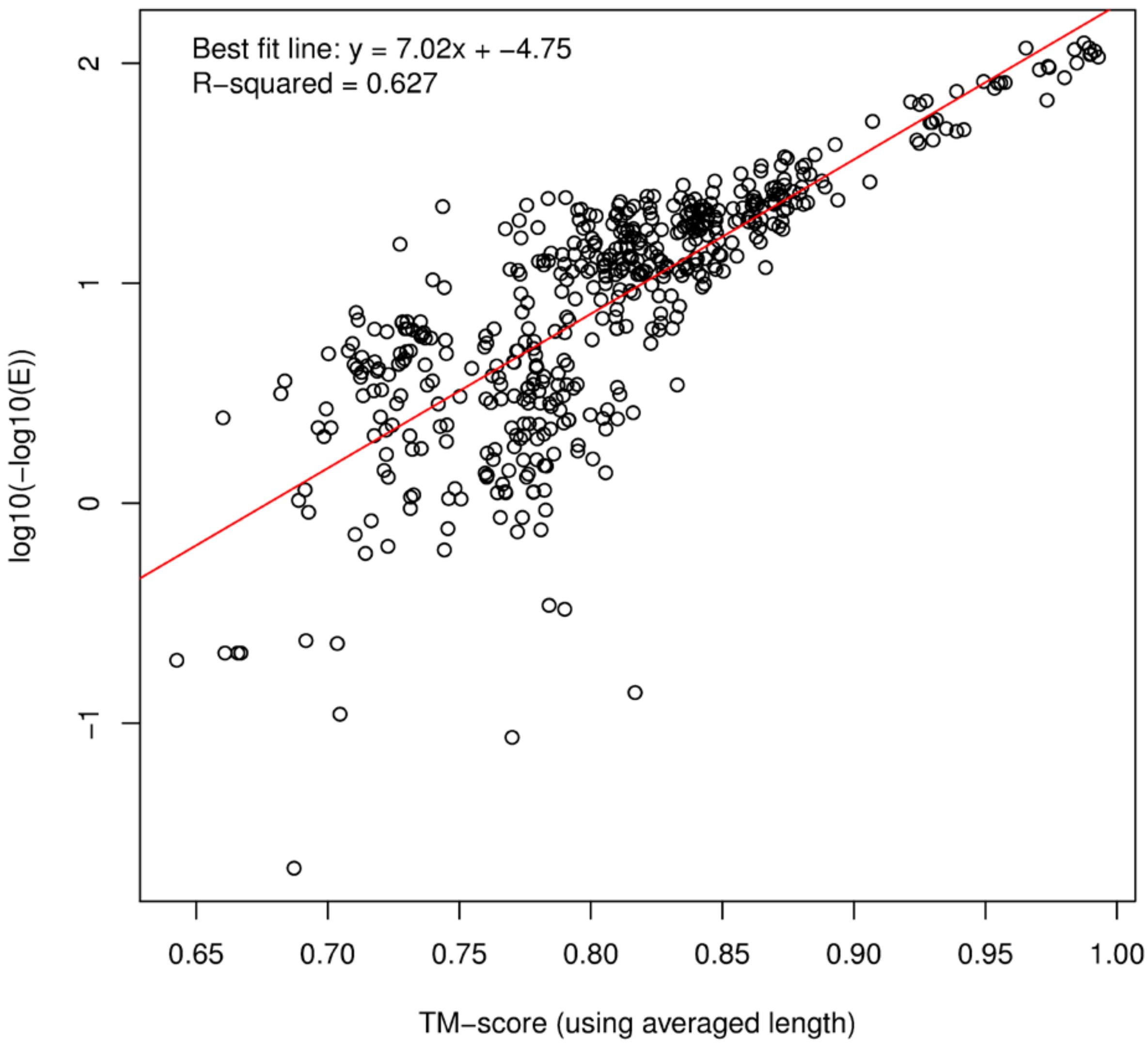

Supplementary Figure 3. Correlation plot between TM-score and log10(-log10(E-value)). Edges between sequence and their associated structure nodes that have both an E-value score < 1 and a TM-align score are plotted and show a roughly linear correlation. This figure is an example of the output from the output_correlation.py Pythoscape plug-in.

Supplemental References

Camacho, C., Coulouris, G., Avagyan, V., Ma, N., Papadopoulos, J., Bealer, K. and Madden T.L. (2009) BLAST+: architecture and applications, *BMC Bioinformatics*, 10, 421.

Li, W. and Godzik, A. (2006) Cd-hit: a fast program for clustering and comparing large sets of protein or nucleotide sequences, *Bioinformatics*, 22, 1658-1659.

Punta, M., Coggill, P.C., Eberhardt, R.Y., Mistry, J., Tate, J., Boursnell, C., Pang, N., Forslund, K., Ceric, G., Clements, J., Heger, A., Holm, L., Sonnhammer, E.L., Eddy, S.R., Bateman, A. and Finn R.D. (2012) The Pfam protein families database, *Nucleic Acids Res*, 40, D290-301.

Zhang, Y., Skolnick J. (2005) TM-align: A protein structure alignment algorithm based on TM-score, Nucleic Acids Research, 33, 2302-2309.
